# Nicotine stimulates ion transport via metabotropic β4 subunit containing nicotinic ACh receptors

**DOI:** 10.1111/bph.15270

**Published:** 2020-11-09

**Authors:** Praveen Kumar, Petra Scholze, Martin Fronius, Gabriela Krasteva‐Christ, Monika I. Hollenhorst

**Affiliations:** ^1^ Institute of Anatomy and Cell Biology Saarland University Homburg Germany; ^2^ Department of Pathobiology of the Nervous System, Center for Brain Research Medical University of Vienna Vienna Austria; ^3^ Department of Physiology and HeartOtago, School of Biomedical Sciences University of Otago Dunedin New Zealand

**Keywords:** ACh receptors, epithelium, nicotine, non‐neuronal cholinergic system, trachea, Ussing chamber

## Abstract

**Background and Purpose:**

Mucociliary clearance is an innate immune process of the airways, essential for removal of respiratory pathogens. It depends on ciliary beat and ion and fluid homeostasis of the epithelium. We have shown that nicotinic ACh receptors (nAChRs) activate ion transport in mouse tracheal epithelium. Yet the receptor subtypes and signalling pathways involved remained unknown.

**Experimental Approach:**

Transepithelial short circuit currents (I_SC_) of freshly isolated mouse tracheae were recorded using the Ussing chamber technique. Changes in [Ca^2+^]_i_ were studied on freshly dissociated mouse tracheal epithelial cells.

**Key Results:**

Apical application of the nAChR agonist nicotine transiently increased I_SC_. The nicotine effect was abolished by the nAChR antagonist mecamylamine. α‐Bungarotoxin (α7 antagonist) had no effect. The agonists epibatidine (α3β2, α4β2, α4β4 and α3β4) and A‐85380 (α4β2 and α3β4) increased I_SC_. The antagonists dihydro‐β‐erythroidine (α4β2, α3β2, α4β4 and α3β4), α‐conotoxin MII (α3β2) and α‐conotoxin PnIA (α3β2) reduced the nicotine effect. Nicotine‐ and epibatidine‐induced currents were unaltered in β2^−/−^mice, but in β4^−/−^ mice no increase was observed. In the presence of thapsigargin (endoplasmatic reticulum Ca^2+^‐ATPase inhibitor) or the ryanodine receptor antagonists JTV‐519 and dantrolene there was a reduction in the nicotine‐effect, indicating involvement of Ca^2+^ release from intracellular stores. Additionally, the PKA inhibitor H‐89 and the TMEM16A (Ca^2+^‐activated chloride channel) inhibitor T16Ainh‐A01 significantly reduced the nicotine‐effect.

**Conclusion and Implications:**

α3β4 nAChRs are responsible for the nicotine‐induced current changes via Ca^2+^ release from intracellular stores, PKA and ryanodine receptor activation. These nAChRs might be possible targets to stimulate chloride transport via TMEM16A.

**What is already known**

- Non‐neuronal nicotinic ACh receptors activate chloride secretion in mouse tracheal epithelium.
- α3β4 nicotinic receptors activate ciliary beat.

**Wha this study adds**

- α3β4 nicotinic receptors are essential for activation of apical chloride secretion in the airways.
- Activation of α3β4 nicotinic receptors mediates Ca^2+^ release from intracellular stores and PKA‐dependent signalling.

**What is the clinical significance**

- α3β4 nAChR‐dependent activation of Ca^2+^‐dependent chloride channel TMEM16A might be beneficial in cystic fibrosis.
- Tracheal nicotinic ACh receptors might serve as possible pharmaceutical targets to stimulate chloride transport.

**Abbreviations**
CaCC: calcium‐activated chloride channel
mAChR: muscarinic ACh receptor
nAChR: nicotinic ACh receptor
RyR: ryanodine receptor

## 1 INTRODUCTION

Strict regulation of transepithelial ion transport is essential for effective mucociliary clearance. Mucociliary clearance represents a crucial primary innate defence mechanism of the airways (Hollenhorst, Richter, & Fronius, 2011; Knowles & Boucher, 2002). This is facilitated by beating of the cilia of ciliated epithelial cells and depending on tightly controlled level and viscosity of the periciliary liquid assured by appropriate regulation of transepithelial ion transport. Several chronic airway diseases may be attributed to impaired mucociliary clearance, including primary ciliary dyskinesia, cystic fibrosis, asthma and chronic obstructive pulmonary disease (COPD) (Dransfield et al., 2013; Knowles & Boucher, 2002). Disruption of transepithelial ion transport is involved in severe pulmonary phenotypes including impaired function of the cystic fibrosis transmembrane conductance regulator in cystic fibrosis, chronic obstructive pulmonary disease and cigarette smoke‐induced chronic bronchitis (Clunes et al., 2012; Dransfield et al., 2013; O'Sullivan & Freedman, 2009).

In previous studies, we found a role for ACh released from non‐neuronal sources as a regulator of airway epithelial ion transport (Hollenhorst, Lips, Weitz, et al., 2012; Hollenhorst, Lips, Wolff, et al., 2012). Since the first studies of the non‐neuronal cholinergic system about 20 years ago (Grando, 1997; Sato et al., 1999; Wessler, Kirkpatrick, & Racké, 1998), it is now well established that ACh plays an important role as autocrine and paracrine signalling molecule in diverse non‐neuronal tissues, including the airway epithelium (Hollenhorst et al., 2020; Perniss, Liu, et al., 2020). ACh transmits its signals via two different classes of receptors: muscarinic (M_1‐5_) receptors and nicotinic receptors (nAChRs) (Lustig, 2006).

In the nervous system, nAChRs are well characterized. Generally, nAChRs form heteropentameric or homopentameric ligand‐gated ion channels, permeable to Na^+^, K^+^ and Ca^2+^ (Wu, Cheng, Jiang, Melcher, & Xu, 2015), that are composed of different subunits (α1–α10, β1–β4, δ, ε and γ) (Lustig, 2006). Considerably less is known about their subunit composition, stoichiometry, function and role in non‐neuronal cells. Several non‐neuronal cells contain nAChR, including epithelial, endothelial, cancer and immune cells (Grando, 1997; Kawashima & Fujii, 2004; Macklin, Maus, Pereira, Albuquerque, & Conti‐Fine, 1998; Medjbera et al., 2015). Yet, in the last decade, there is increasing evidence that nAChRs may additionally exert metabotropic actions, as they increase the intracellular Ca^2+^ concentration without forming Ca^2+^‐permeable ion channels in T cells (Razani‐Boroujerdi et al., 2007), modulate ATP‐induced Ca^2+^ release in rat alveolar macrophages (Mikulski et al., 2010) and activate the Na^+^/K^+^ATPase via a protein kinase C (PKC)‐dependent pathway in rat colonic epithelium (Lottig, Bader, Jimenez, & Diener, 2019). Additionally, metabotropic nAChRs were found in monocytes (Richter et al., 2016) and airway epithelial cell lines, where their activation inhibited ATP‐mediated release of IL‐1β (Richter et al., 2018).

In mouse tracheal epithelium, nicotine binding to apical nAChRs activates apical chloride secretion driven by basolateral potassium secretion (Hollenhorst, Lips, Weitz, et al., 2012). However, the nAChR subtype, the ionotropic or metabotropic nature of these receptors and the ion channel subtypes facilitating the secretion of Cl^−^ are unknown. Here, we aim to identify the nAChR subtype as well as the nAChR‐activated channels responsible for the ion transport changes and discuss whether this is a suitable target to activate chloride secretion to restore mucociliary clearance.

## 2 METHODS

### 2.1 Compliance with requirements for studies using animals

Animal studies are reported in compliance with the ARRIVE guidelines (Percie du Sert et al., 2020) and with the recommendations made by the *British Journal of Pharmacology* (Lilley et al., 2020). All animal care and experimental procedures were approved by the German and European animal welfare committee and performed according to the German and European animal welfare law. Adult wild‐type (WT, C57Bl/6J, RRID:IMSR_JAX:000664) and β2^−/−^ (Picciotto et al., 1995) or β4^−/−^ nAChR mice (Kedmi, Beaudet, & Orr‐Urtreger, 2004) aged between 10 and 15 weeks of both sexes were used throughout the study. The β2^−/−^ mice were backcrossed to C57Bl/6J for 12 generations and the β4^−/−^ mice were backcrossed to C57Bl/6J for six generations after germline transmission. The animals were randomly chosen once they had the appropriate age. The WT mice were bred and housed in IVC cages in the animal facility of the Institute of Experimental Surgery of the Saarland University under standardized 12‐h day–night cycles with free access to food and water. β2^−/−^ or β4^−/−^ nAChR mice were bred and held under specific pathogen‐free conditions in the animal facility of the Medical University of Vienna. After shipping, these mice were housed in the animal facility of the Institute of Experimental Surgery of the Saarland University for 2 weeks for acclimatization and quarantine before the experiments were performed.

### 2.2 Materials

d‐glucose, calcium d‐gluconate, DMSO, ACh, ATP, mecamylamine, NaHCO_3_, papaine, l‐cysteine, leupeptine, pyruvic acid, NaOH and tyrphostin AG490 (AG 490) were acquired from Sigma‐Aldrich (Taufkirchen, Germany). A‐85380, α‐bungarotoxin, α‐conotoxin PnIA, α‐conotoxin MII, chelerythrine chloride, dihydro‐β‐erythroidine (DHβE), epibatidine, T16Ainh‐A01, Ani 9, CaCCinh‐A01, Chromanol293B, ACV 1 (conotoxin Vc1.1), dantrolene, JTV519 and H‐89 were obtained from Tocris Bioscience (Abingdon, UK). The α‐conotoxin ImI was ordered from Alomone Labs (Jerusalem, Israel). KH_2_PO_4_, KCl and MgCl_2_ were purchased from MERCK (Darmstadt, Germany). NaCl was ordered from Grüssing GmbH (Westoverledingen, Germany). HEPES and BSA were from Carl Roth (Karlsruhe, Germany). Thapsigargin and WP1066 were acquired from Cayman Chemicals (Ann Arbor, MI, USA) and nicotine from Glentham Life Sciences (Corsham, UK). EDTA and Tween 20 were obtained from VWR (Darmstadt, Germany). Sodium pyruvate was purchased from Gibco (Thermo Fisher Scientific, Waltham, MA, USA) and DNase1 from Invitrogen (Thermo Fisher Scientific).

### 2.3 Ussing chamber experiments

For Ussing chamber measurements, mice were exposed to an overdose of the narcotic isoflurane and killed by aortic exsanguination. The WT animals used for experiments with different agonists and antagonists were randomly chosen. Experiments in which the responses in WT animals were compared to knockout (KO) animals were performed blinded. The trachea was immediately dissected and the surrounding connective tissue was removed. Then the freshly isolated tracheae were longitudinally opened by cutting the ventral midline under the dissection microscope and mounted into a modified Ussing chamber with a circular aperture of 1.8 mm^2^ as described previously (Hollenhorst, Lips, Wolff, et al., 2012). The tissue was continuously perfused with buffer solution heated to 37°C which had the following composition (in mmol·L^−1^):‐ 1.3 Ca^2+^ gluconate, 145 NaCl, 5 d‐glucose, 5 HEPES, 1 MgCl_2_ and 2 KH_2_PO_4_ (pH 7.4). The Ag^+^/AgCl electrodes were linked with bridges of 2% agar and 3 mol·L^−1^ KCl to the compartments of the Ussing chamber. The spontaneously generated transepithelial potential (V_T_) was clamped to 0 V with a voltage‐clamp amplifier (KU Leuven, Belgium) after an equilibration period of approximately 5 min. The short circuit current (I_SC_) was continuously recorded via a PowerLab version 4/35 (ADInstruments, Spechbach, Germany) with LabChart version 8 (ADInstruments). All substances were administered after an equilibration period of about 20 min. To assure tissue viability, ATP (100 μmol·L^−1^) was applied to the apical side of the tissue at the end of each experiment. Tissues that did not respond to ATP were not considered for data analyses.

### 2.4 RT‐PCR experiments

For RT‐PCR experiments, the tracheal epithelium of WT mice was scraped with a sterile cotton swab. The RNeasy Mini Kit (Qiagen, Hilden, Germany) was used for RNA extraction according to the manufacturer's instructions. Contaminating DNA was removed by adding DNase‐I (Invitrogen, Karlsruhe, Germany). Reverse transcription of the RNA was performed using the superscript II reverse transcriptase (Invitrogen) for 50 min at 42°C and the cDNA was amplified with specific intron‐spanning primers (MWG, Ebersberg, Germany, Table 1). Twenty‐five microlitres of RT‐PCR preparations consisting of 2.5‐μl buffer II, 2‐μl MgCl_2_ (25 mmol·L^−1^), 0.625‐μl dNTP (10 mmol·L^−1^), 0.625‐μl primer (20 pmol·L^−1^), 0.125‐μl AmpliTaq Gold polymerase (5 U·μl^−1^, Applied Biosystems, Branchburg, NJ, USA) and 2‐μl cDNA supplemented with RNase‐free H_2_O were subjected an initial denaturation of 12 min at 95°C followed by cycles of 20 s at 95°C, 45 s at a primer‐specific annealing temperature of 60°C, 20 s at 72°C and a final extension of 7 min at 72°C. Separation of the PCR products was performed on a 1.25% TRIS‐acetate‐EDTA gel.

**TABLE 1:** Sequences of the primers used for RT‐PCR experiments of the tracheal epithelium

nAChR subunit	Accession no.	Sequence	Product length (bp)
α2	NM_144803	fwd: ctcccatcctgctttccag	115
		rev: gtttgaacaggcggtcctc	
α3	NM_145129	fwd: cgcctgttccagtacctgtt	196
		rev: cagagggtttccatttcagc	
α4	NM_015730	fwd: ctcagatgtggtccttgtcc	238
		rev: ggtgggtgactgcaaagttc	
α5	NM_176844	fwd: ccagctaatgaccaccaacg	218
		rev: gctgcgtccaagtgacagt	
α6	NM_021369	fwd: cctgcactgcggtttatgtc	231
		rev: cagccacagattggtctcca	
α7	NM_007390	fwd: acaatacttcgccagcacca	144
		rev: aaaccatgcacaccagttca	
α9	NM_001081104	fwd: caatgctctgcgtccagtag	208
		rev: acaccagatcgctgggaa	
α10	NM_001081424	fwd: tctgctcctgctctttctcc	207
		rev: ccacaggtacaaggtcagca	

### 2.5 Calcium imaging experiments

For isolation of tracheal epithelial cells for calcium imaging experiments, randomly chosen mice were killed as described above. Then the mouse tracheae were dissected into rings and transferred into 1‐ml digestion media containing 1‐ml Tyrode's I solution consisting of (in mmol·L^−1^) 140 NaCl, 10 glucose, 10 HEPES, 5 KCl, 1 MgCl_2_, 1 sodium pyruvate, 5 NaHCO_3_, supplemented with 2 mg of papaine (10 U·mg^−1^), 12 μmol·L^−1^ l‐cysteine, 0.5 μl of DNase1 and 4 μmol·L^−1^ EDTA. The rings were digested for 40 min in a water bath at 37°C. Then they were removed from the solution and the solution was centrifuged for 5 min at 900 *g*. The supernatant was removed and the cell pellet resuspended in stop solution containing 1‐ml Tyrode's II (in mmol·L^−1^: 140 NaCl, 10 HEPES, 10 glucose, 5 KCl, 1 MgCl_2_, 1 CaCl_2_, 1 sodium pyruvate and 1 pyruvic acid) and 2 μl of 10 mg·ml^−1^ leupeptin. The mix was kept for 5 min at room temperature and again centrifuged for 5 min at 900 *g*. The supernatant was removed and the cells were collected in 90‐μl Tyrode's III buffer consisting of (in mmol·L^−1^) 130 NaCl, 10 glucose, 10 HEPES, 5 KCl, 8 CaCl_2_, 1 MgCl_2_, 10 sodium pyruvate and 5 NaHCO_3_. Thirty microlitres of the cell suspension was added on a sterile coverslip coated with 285 μl of 0.1 mol·L^−1^ sodium bicarbonate, 5 μl of 1‐N NaOH and 10 μl of Cell‐Tak (Corning, New York, USA). Cells were incubated for 35–45 min at 37°C on the coverslips and afterwards loaded with 100‐μl Tyrode's III containing 4‐μl Fura‐2‐AM (Invitrogen) and 2‐μl pluronic (Sigma‐Aldrich) for 30 min at 37°C. Before the start of the measurements, coverslips were washed two times with Tyrode's III. Fura‐2‐AM in the loaded cells was excited with a 340/380‐nm ratio of light from a DG4 wavelength switching xenon arc lamp (Sutter Instruments, Novato, California, USA). For fluorescence detection, an Orca Flash 4.0 camera was used (Hamamatsu, Herrsching, Germany). Analyses were performed with the NIS‐Elements software (Nikon Instruments, Amsterdam, Netherlands).

### 2.6 Experimental design and statistical analysis

The data and statistical analysis comply with the recommendations of the *British Journal of Pharmacology* on experimental design and analysis in pharmacology (Curtis et al., 2018). In the Ussing chamber experiments, the values were calculated for 1‐cm^2^ tissue area and reported as mean ± SEM with the number (*n*) of the investigated tracheas and animals. In all Ussing chamber experiments, a group size of *n* = 5–7 animals was designed. According to our previous experience, this was identified as a suitable size to evaluate statistical significances (Hollenhorst, Lips, Kummer, & Fronius, 2012; Hollenhorst, Lips, Weitz, et al., 2012; Hollenhorst, Lips, Wolff, et al., 2012). For the calcium imaging experiments, coverslips with cells isolated from at least five different animals were measured with “*N*” denoting the number of animals and “*n*” the number of cells evaluated. All data were first analysed for normal distribution with the Kolmogorov–Smirnov test. Afterwards, the paired or unpaired Student's *t*‐test was applied when data passed the normality test. Datasets that did not pass the normality test were analysed with the Mann–Whitney *U* test. Statistical significance was assigned for *P <* 0.05.

### 2.7 Nomenclature of targets and ligands

Key protein targets and ligands in this article are hyperlinked to corresponding entries in the IUPHAR/BPS Guide to PHARMACOLOGY http://www.guidetopharmacology.org and are permanently archived in the Concise Guide to PHARMACOLOGY 2019/20 (Alexander, Fabbro, et al., 2019, Alexander, Mathie, et al., 2019).

## 3 RESULTS

### 3.1 Nicotine activates transepithelial ion current in WT mice

**FIGURE 1:**
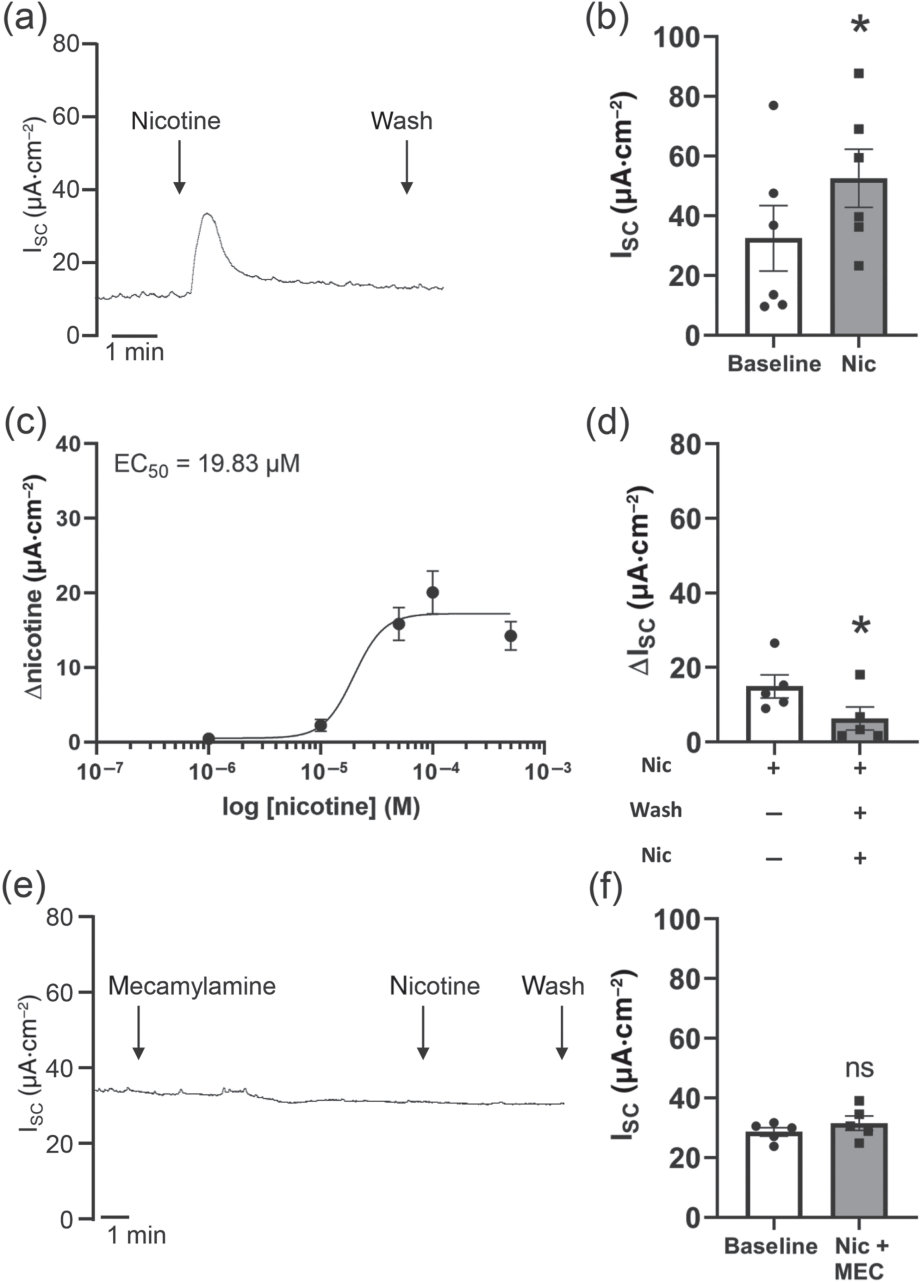
Effect of nicotine on transepithelial ion transport of mouse tracheal epithelium. (a) Apical application of nicotine (100 μmol·L^−1^) resulted in a transient increase in short circuit current (I_SC_). Representative current trace. (b) The nicotine‐induced current increase was significant compared to baseline (*n* = 5, *P < 0.05). (c) The nicotine effect was dose dependent (*n* = 5 for each concentration). (d) Repeated apical nicotine application (100 μmol·L^−1^) in the same trachea showed a reduced nicotine‐activated current (ΔI_SC_) upon the second application (*n* = 5, *P < 0.05). (e) Mecamylamine (25 μmol·L^−1^, apical) abolished the nicotine‐induced I_SC_. Application of mecamylamine did not influence I_SC_. Representative current trace. (f) In the presence of the non‐selective nAChR antagonist mecamylamine (MEC, 25 μmol·L^−1^, apical), nicotine had no significant effect on I_SC_ (*n* = 5; ns, not significant)

First, we characterized the basic effect of nicotine on transepithelial ion transport. We applied nicotine on the apical side of the epithelium, in order to investigate possible non‐neuronal effects of nAChRs. Apical application of 100 μmol·L^−1^ nicotine transiently increased I_SC_ (20.09 ± 2.89 μA·cm^−2^, Figure 1a,b). The nicotine effect was dose dependent with an EC_50_ of 19.83 μmol·L^−1^ (Figure 1c). When nicotine (100 μmol·L^−1^, apical) was applied two times repeatedly, the second nicotine‐induced peak was significantly smaller (of in total 6.11 ± 3.11 μA·cm^−2^, Figure 1d), indicating a desensitization. The nicotine effect was abolished by the general nAChR antagonist mecamylamine (25 μmol·L^−1^, reduction of 85.35%, Figure 1e,f), indicating that the nicotine‐induced effect was indeed due to an activation of nAChRs.

### 3.2 Nicotine acts on heteromeric αβ nAChR

In the murine tracheal epithelium, we previously detected transcripts for several nAChR subunits, comprising α3, α4, α5, α7, α9, α10, β2 and β4 (Hollenhorst, Lips, Weitz, et al., 2012). However, it remained elusive, which receptor subtype is responsible for the observed ion transport changes. Therefore, we here analysed the abundancy of the different α nAChR subunits in every single epithelium of 18 different mice via RT‐PCR. The α3 and α10 subunits were present in 17 out of 18 epithelia, making them highly probable candidates for being involved in the nicotine effect. Also, the α4 subunit with 14 out of 18 epithelia and the α7 subunit with 12 out of 18 epithelia showed high distribution, while the α5 and α9 subunits were present only partially (5 of 18 and 7 of 18, respectively) making them less likely candidates (Table 2).

**TABLE 2:** Different α nicotinic ACh receptor subunits detected with RT‐PCR in mouse tracheal epithelium

Mouse	α2	α3	α4	α5	α6	α7	α9	α10
1	‐	x	x	‐	‐	x	x	x
2	‐	x	‐	‐	‐	x	‐	x
3	‐	x	x	x	‐	x	x	‐
4	x	x	x	‐	‐	x	x	x
5	‐	x	x	‐	‐	x	‐	x
6	‐	x	x	‐	‐	x	‐	x
7	‐	x	x	x	‐	x	‐	x
8	x	x	x	‐	‐	x	x	x
9	‐	x	x	‐	‐	x	‐	x
10	‐	x	x	x	‐	x	x	x
11	‐	x	x	x	‐	‐	x	x
12	‐	x	x	‐	‐	‐	‐	x
13	‐	x	x	‐	‐	‐	‐	x
14	‐	x	x	x	x	‐	x	x
15	‐	x	x	‐	‐	‐	‐	x
16	‐	‐	‐	‐	‐	x	‐	x
17	‐	x	‐	‐	‐	x	‐	x
18	‐	x	‐	‐	‐	‐	‐	x
Total	2	17	14	5	1	12	7	17

**FIGURE 2:**
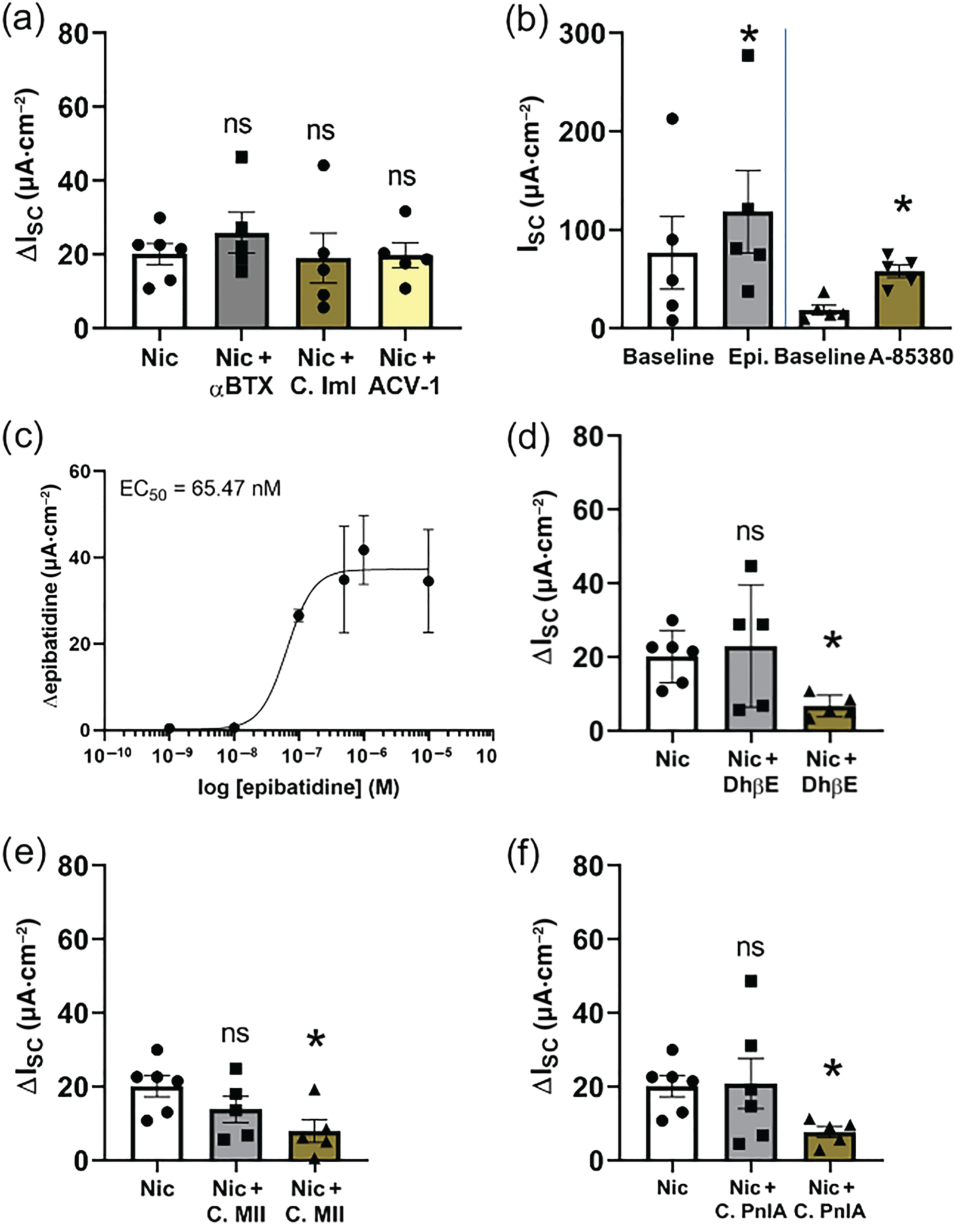
Effect of nicotinic receptor agonists and antagonists on transepithelial ion transport of mouse tracheal epithelium. (a) The α7 nicotinic ACh receptor (nAChR) antagonist α‐bungarotoxin (αBTX, 100 nmol·L^−1^, *n* = 5, apical) or the α7 nAChR antagonist α‐conotoxin ImI (C. ImI, 4 μmol·L^−1^, apical) or the α9α10 nAChR antagonist ACV‐1 (100 nmol·L^−1^, apical) did not influence the nicotine effect (100 μmol·L^−1^, apical, ΔI_SC_; ns, not significant). (b) The epibatidine‐induced (α4β2, α3β2, α4β4 and α3β4 nAChR agonist) current peak (1 μmol·L^−1^) was significant compared to baseline current (*n* = 5, *P < 0.05). Application of the α4β2 and α3β4 nAChR agonist A‐85380 (100 μmol·L^−1^, apical, *n* = 5) significantly increased I_SC_ (*P < 0.05). (c) The epibatidine effect was dose dependent (*n* = 5 for each concentration). (d) In the presence of 1 μmol·L^−1^ of the α4β2, α3β2, α4β4 and α3β4 nAChR antagonist dihydro‐β‐erythroidine (DhβE), the nicotine effect (ΔI_SC_) was similar to control conditions (*n* = 5; ns, not significant), but 10 μmol·L^−1^ DhβE significantly reduced the nicotine‐induced current (ΔI_SC_, *n* = 5, *P < 0.05). (e) The α3β2 nAChR antagonist α‐conotoxin MII (C. MII) did not influence the nicotine effect (ΔI_SC_) in a concentration of 50 nmol·L^−1^ (*n* = 5; ns, not significant) but significantly reduced the nicotine‐induced current (ΔI_SC_, *n* = 5, *P < 0.05) in a concentration of 1 μmol·L^−1^. (f) The α3β2 antagonist α‐conotoxin PnIA (C. PnIA) did not influence the nicotine effect (ΔI_SC_) in a concentration of 100 nmol·L^−1^ (*n* = 6; ns, not significant) but significantly reduced the nicotine‐induced current in a concentration of 500 mmol·L^−1^ (ΔI_SC_, *n* = 5, *P < 0.05)

In the presence of the antagonist for solely α subunit containing nAChRs (α7, α9 and α9α10, McIntosh, Absalom, Chebib, Elgoyhen, & Vincler, 2009) α‐bungarotoxin (100 nmol·L^−1^), the nicotine effect (100 μmol·L^−1^, apical, at supramaximal dose) did not differ from control conditions (Figure 2a). Additionally, in the presence of the α7 nAChR antagonist α‐conotoxin IMI (1 μmol·L^−1^, apical) as well as the α9α10 nAChR antagonist ACV 1 (100 nmol·L^−1^, apical), the nicotine effect was similar to control conditions (Figure 2a). Taken together, these results indicate that neither α7, α9 nor α9α10 are involved in the nicotine effect. Therefore, we next investigated the involvement of mixed αβ heteromeric nAChRs in the nicotine effect and used different agonists and antagonists to identify possible α subunits. The α4β2 and α3β4 agonist A‐85380 (100 μmol·L^−1^, Sullivan et al., 1996; Whiteaker et al., 2002) led to a significant transient I_SC_ increase (39.27 ± 8.49 μA·cm^−2^, Figure 2b) that was almost twice as large as the nicotine effect. Similarly, epibatidine (1 μmol·L^−1^), an α3β2, α4β2, α4β4 and α3β4 nAChR agonist (Chavez‐Noriega et al., 1997; Stauderman et al., 1998) applied apically, led to a significant transient increase in I_SC_ (41.76 ± 8.02 μA·cm^−2^, Figure 2b). This effect was dose dependent with an EC_50_ of 65.47 nmol·L^−1^ (Figure 2c), indicating that the activated nAChRs were more sensitive to epibatidine than to nicotine. In the presence of 1 μmol·L^−1^ dihydro‐β‐erythroidine, an antagonist for α4β4, α4β2, α3β2 and α3β4 nAChRs with decreasing affinity (Harvey & Luetje, 1996), the nicotine effect was similar to control conditions (Figure 2d). Application of 10 μmol·L^−1^ dihydro‐β‐erythroidine reduced the nicotine effect by 69.01% (Figure 2d), hinting to an involvement of α3 containing nAChRs rather than α4 containing nAChRs in the nicotine effect. The α3β2 antagonist α‐conotoxin MII (Cartier et al., 1996) did not influence the nicotine effect at a concentration of 50 nmol·L^−1^ (Figure 2e), while it significantly reduced the nicotine effect (by 60.56%) in a concentration of 1 μmol·L^−1^ (Figure 2e). Another α3β2 antagonist, α‐conotoxin PnIA (Luo et al., 1999), did not influence the nicotine effect at a concentration of 100 nmol·L^−1^, when applied apically (Figure 2f), but at a concentration of 500 nmol·L^−1^, the nicotine effect was significantly reduced by 61.69% (Figure 2f). This further indicates that nAChR responsible for the nicotine effect contains the α3 subunit.

**FIGURE 3:**
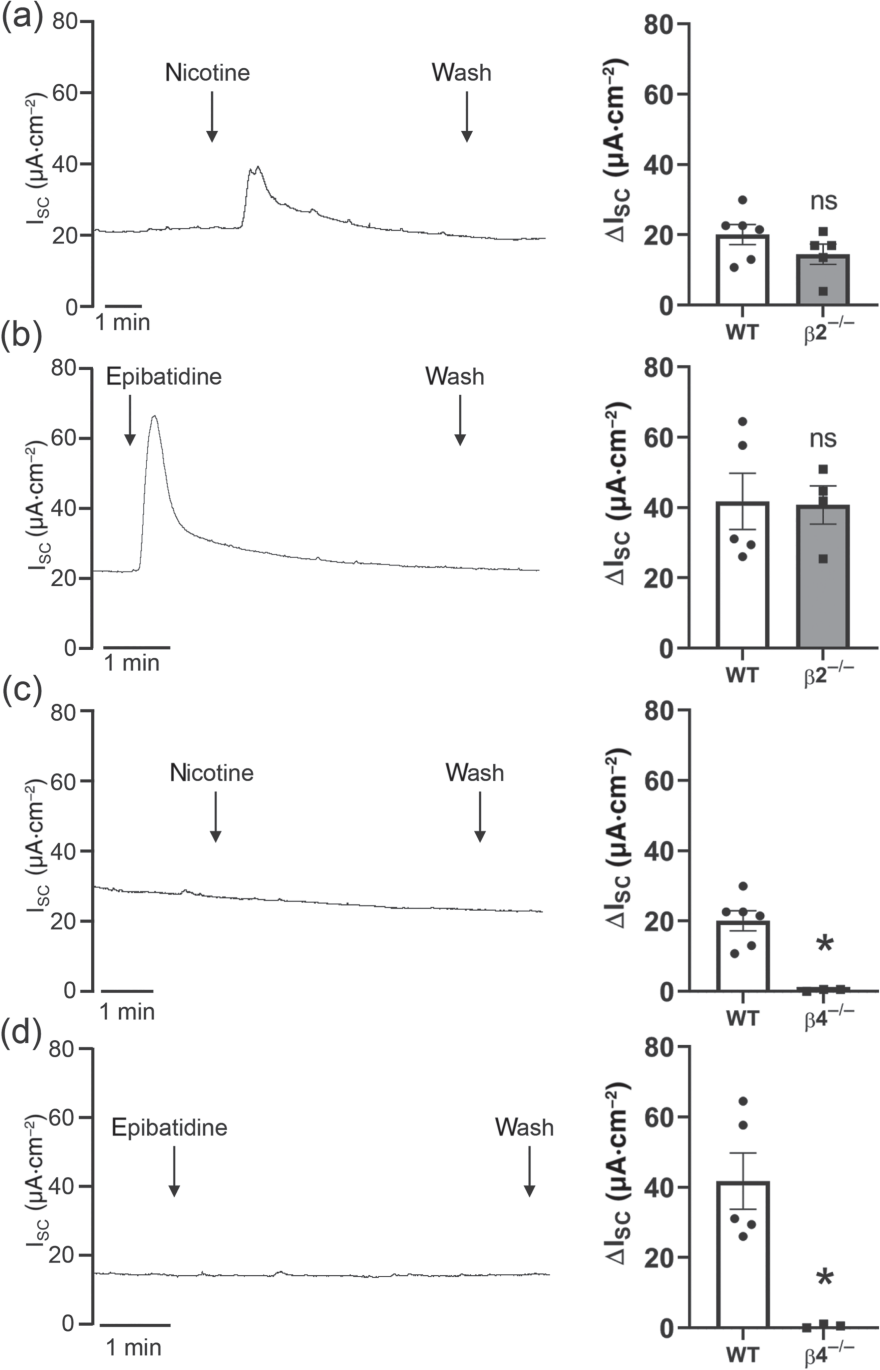
Effect of nicotine or epibatidine on transepithelial ion transport in β2‐ or β4‐deficient mice. (a) Apical application of nicotine (100 μmol·L^−1^) in β2^−/−^ mice resulted in a transient reversible increase in the short circuit current (I_SC_). Representative current trace. (b) The nicotine‐induced peak (ΔI_SC_) in β2^−/−^ mice was similar to wild‐type (WT) mice (*n* = 5; ns, not significant). (c) Apical application of epibatidine (1 μmol·L^−1^) in β2^−/−^ mice resulted in a transient reversible increase in I_SC_. Representative current trace. (d) The epibatidine‐induced peak (ΔI_SC_) was similar in WT and β2^−/−^ mice (*n* = 5; ns, not significant). (e) Apical application of nicotine (100 μmol·L^−1^) in β4^−/−^ mice did not influence I_SC_. Representative current trace. (f) The nicotine‐induced peak (ΔI_SC_) in β4^−/−^ mice was significantly reduced compared to WT mice (*n* = 5, *P < 0.05). (g) Apical application of epibatidine (1 μmol·L^−1^) in β4^−/−^ mice had no effect on I_SC_. Representative current trace. (h) The epibatidine‐induced peak (ΔI_SC_) was significantly reduced in β4^−/−^ mice compared to WT mice (*n* = 5, *P < 0.05)

For characterization of the β subunit involved in the nicotine‐induced increase of the transepithelial ion current in the mouse tracheal epithelium, Ussing chamber experiments were performed with β2^−/−^ and β4^−/−^ nAChR mice. In β2^**−/−**^ mice, the nicotine‐induced current (100 μmol·L^−1^, apical) and the current induced by epibatidine (1 μmol·L^−1^, apical) were similar to WT mice (Figure 3a–d). In contrast to this, in β4^−/−^ mice, the effect on ion transport induced by 100 μmol·L^−1^ nicotine and by 1 μmol·L^−1^ epibatidine, both applied apically, was completely abolished (Figure 3e–h), demonstrating that the nAChR responsible for the ion transport changes contains the β4 subunit and that this subunit is essential for nicotine effect.

### 3.3 The nicotine effect is associated with Ca^2+^‐ and PKA‐dependent intracellular signalling

**FIGURE 4:**
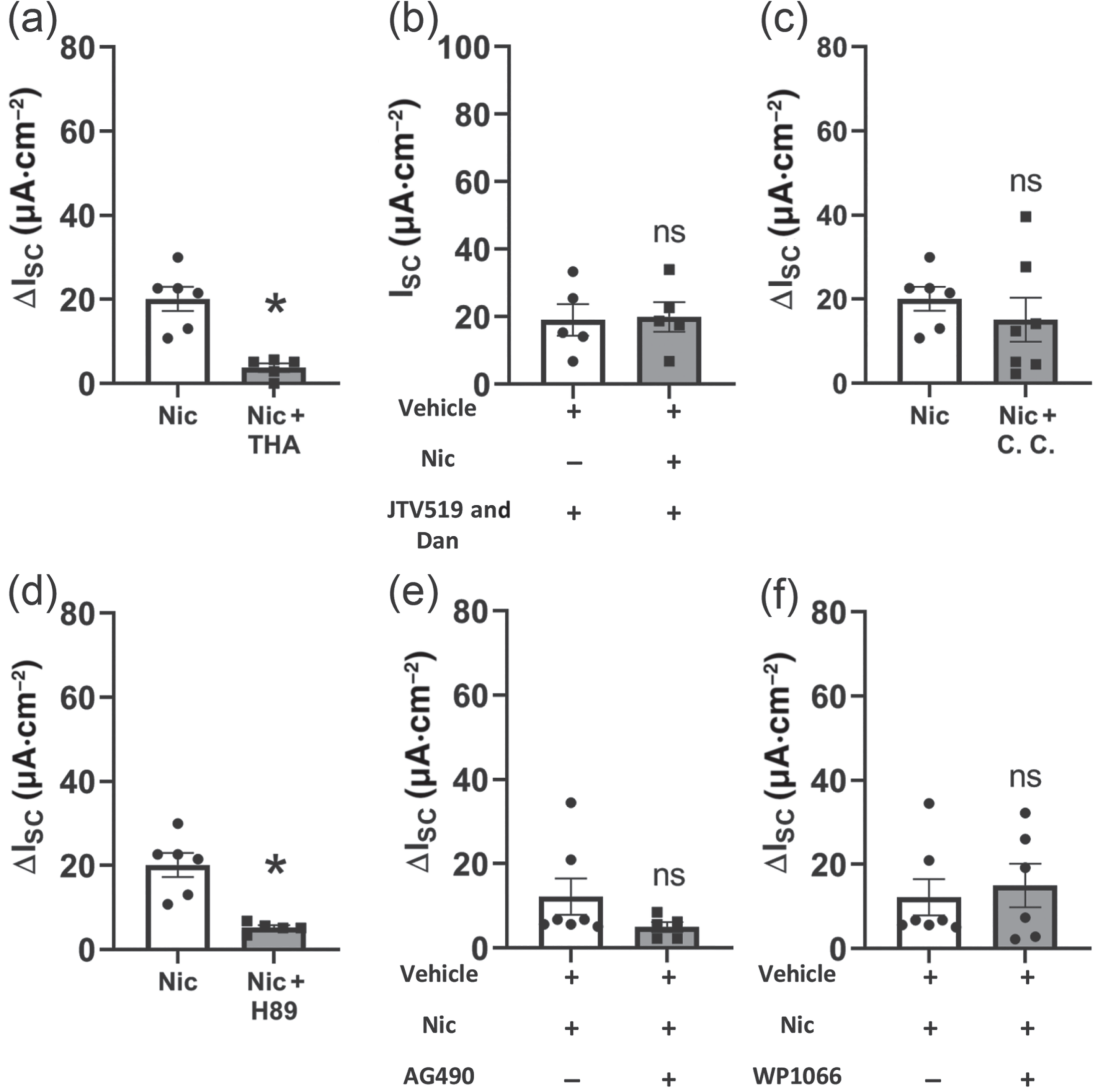
Downstream signalling involved in the nicotine‐induced activation of transepithelial ion transport in mouse trachea. (a) Application of 1‐μM thapsigargin (THA, apical, *n* = 5), an inhibitor of the Ca^2+^‐ATPase in the endoplasmatic reticulum, significantly reduced the nicotine effect (100 μM, apical, ΔI_SC_, *P < 0.05). (b) In the presence of the ryanodine receptor antagonist JTV519 and dantrolene (each 10 μmol·L^−1^, apical), nicotine (100 μmol·L^−1^, apical) had no effect on I_SC_ (ns, not significant; *n* = 5). (c) The protein kinase C (PKC) inhibitor chelerythrine chloride (CC, 5 μmol·L^−1^, apical and basolateral, *n* = 7) did not influence the nicotine effect (100 μmol·L^−1^, apical, ΔI_SC_; ns, not significant). (d) In the presence of the protein kinase A (PKA) inhibitor H‐89 (10 μmol·L^−1^, apical and basolateral, *n* = 5), the current induced by nicotine (100 μmol·L^−1^, apical, ΔI_SC_, *P < 0.05) was significantly reduced. (e) Application of the JAK2 inhibitor AG 490 (50 μmol·L^−1^, apical and basolateral, *n* = 5) did not lead to a significant change of the nicotine effect (100 μmol·L^−1^, apical, ΔI_SC_, *P < 0.05). (f) Application of the STAT3 inhibitor WP1066 (10 μmol·L^−1^, apical, *n* = 6) did not influence the nicotine effect (100 μM, apical, ΔI_SC_; ns, not significant)

To investigate if activation of tracheal epithelial nAChR leads to a release of Ca^2+^ from intracellular stores, we performed experiments with thapsigargin, an inhibitor of the Ca^2+^ ATPase in the endoplasmatic reticulum (Thastrup, Cullen, Drobak, Hanley, & Dawson, 1990). In the presence of 1 μmol·L^−1^ thapsigargin, applied apically, the nicotine effect was significantly reduced by 81.40% (Figure 4a), indicating that the activation of the nicotine‐induced ion current changes is due to a release of Ca^2+^ from intracellular stores. One signalling pathway leading to a release of Ca^2+^ from intracellular stores, such as the endoplasmatic reticulum, is mediated by ryanodine receptors (RyR1‐3, Gerasimenko et al., 2006). Therefore, we next aimed to elucidate if RyR are responsible for the nicotine effect in mouse tracheal epithelium. We applied the antagonists JTV519 (10 μmol·L^−1^) and dantrolene (10 μmol·L^−1^) in order to inhibit all RyR1‐3 subtypes. Simultaneous application of these antagonists led to the nicotine effect being completely abolished (Figure 4b). Because an increase in intracellular Ca^2+^ may also activate other effector proteins, such as the PKC which in turn is able to act on ion channels (Cui et al., 2019; Wang et al., 2019), we investigated the influence of PKC on the nicotine‐induced ion current. Application of the PKC inhibitor chelerythrine chloride (5 μmol·L^−1^, apical and basolateral, Herbert, Augereau, Gleye, & Maffrand, 1990) did not affect the nicotine‐induced current (Figure 4c). This indicates that Ca^2+^ released through RyR does not activate PKC and, thus, PKC‐activated transport proteins.

Because Ca^2+^‐ and cAMP‐dependent signalling pathways may be interconnected, we further investigated the involvement of cAMP‐dependent signalling on the nicotine effect. Inhibition of the PKA with the inhibitor H‐89 (10 μmol·L^−1^, apical and basolateral) significantly reduced the nicotine effect by 74.08% (Figure 4d), indicating a role for cAMP‐dependent PKA activation.

Because some nAChRs have been shown to be coupled to the JAK2–STAT3‐dependent pathway before eliciting effects further downstream (de Jonge et al., 2005; Hosur & Loring, 2011), we investigated if this signalling pathway was also involved in the nicotine effect in the signalling cascade before the release of Ca^2+^ from intracellular stores. Application of the JAK2 inhibitor AG 490 (50 μmol·L^−1^, apical and basolateral, Meydan et al., 1996) or the STAT3 inhibitor WP1066 (10 μmol·L^−1^, apical, Hussain et al., 2007) did not lead to a significant change of the nicotine effect ( Figure 4e,f), indicating that the JAK2–STAT3 signalling pathway is not involved in the nicotine‐induced current changes.

### 3.4 The nicotine effect is mainly mediated by Ca^2+^ release from intracellular stores

**FIGURE 5:**
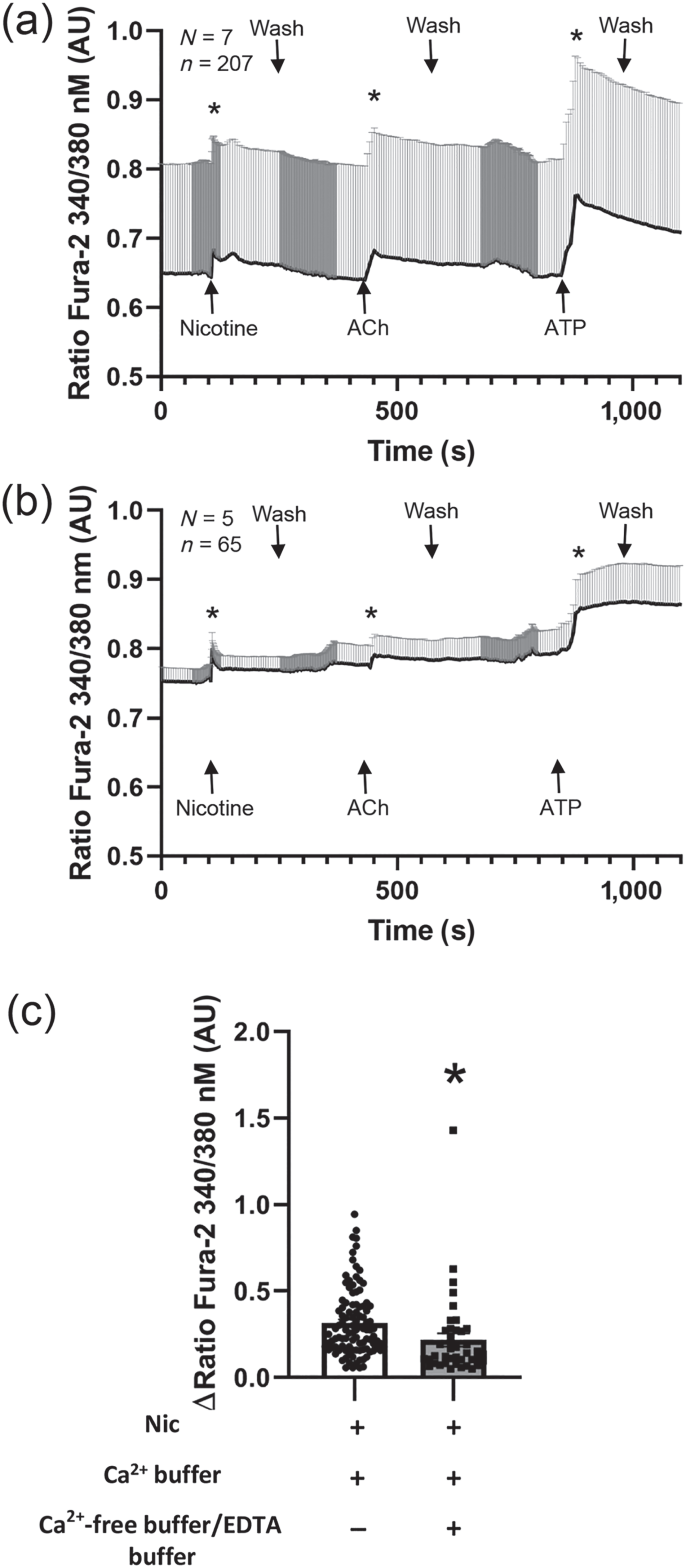
Ca^2+^ imaging and immunohistochemistry experiments of tracheal epithelial cells of wild‐type mice. (a) Application of nicotine, ACh, or ATP significantly increased [Ca^2+^]_i_ compared to baseline in the presence of Ca^2+^‐containing external solution (normal Tyrode's). *N*, number of animals; *n*, number of cells. (b) Application of nicotine, ACh, or ATP increased [Ca^2+^]_i_ compared with baseline in the presence of Ca^2+^‐free external solution. *N*, number of animals; *n*, number of cells. (c) The nicotine‐induced [Ca^2+^]_i_ was significantly reduced in Ca^2+^‐free buffer compared to extracellular Ca^2+^‐containing solution (Tyrode's)

To clarify if the nicotine effect involves Ca^2+^‐permeable nAChRs or metabotropic‐mediated release of Ca^2+^ from intracellular stores, we performed Ca^2+^ imaging experiments on freshly isolated mouse tracheal epithelial cells. Experiments were carried out with Ca^2+^‐containing Tyrode's medium or Ca^2+^‐free EGTA‐containing Tyrode's medium in order to achieve extracellular Ca^2+^‐free conditions (Figure 5a,b). In both cases, a significant increase in [Ca^2+^]_i_ was observed in the presence of nicotine, although the increase was significantly smaller in Ca^2+^‐free Tyrode's (Figure 5c). This indicates that the nAChRs in the tracheal epithelium are mainly, but not completely, increasing [Ca^2+^]_i_ via Ca^2+^ release from intracellular stores rather than via an influx of Ca^2+^ ions through the nAChRs themselves or via other mechanisms such as voltage‐gated Ca^2+^ channels in the plasma membrane. In addition, it was noted that the same percentage of cells (41%) were reacting to nicotine in experiments with and without extracellular Ca^2+^, indicating that while the amplitude of the reaction was reduced in Ca^2+^‐free conditions, it did not affect the total number of reacting cells.

### 3.5 The chloride channel TMEM16A and the potassium channel KCNQ1(K_v_7.1) are involved in the nicotine effect

**FIGURE 6:**
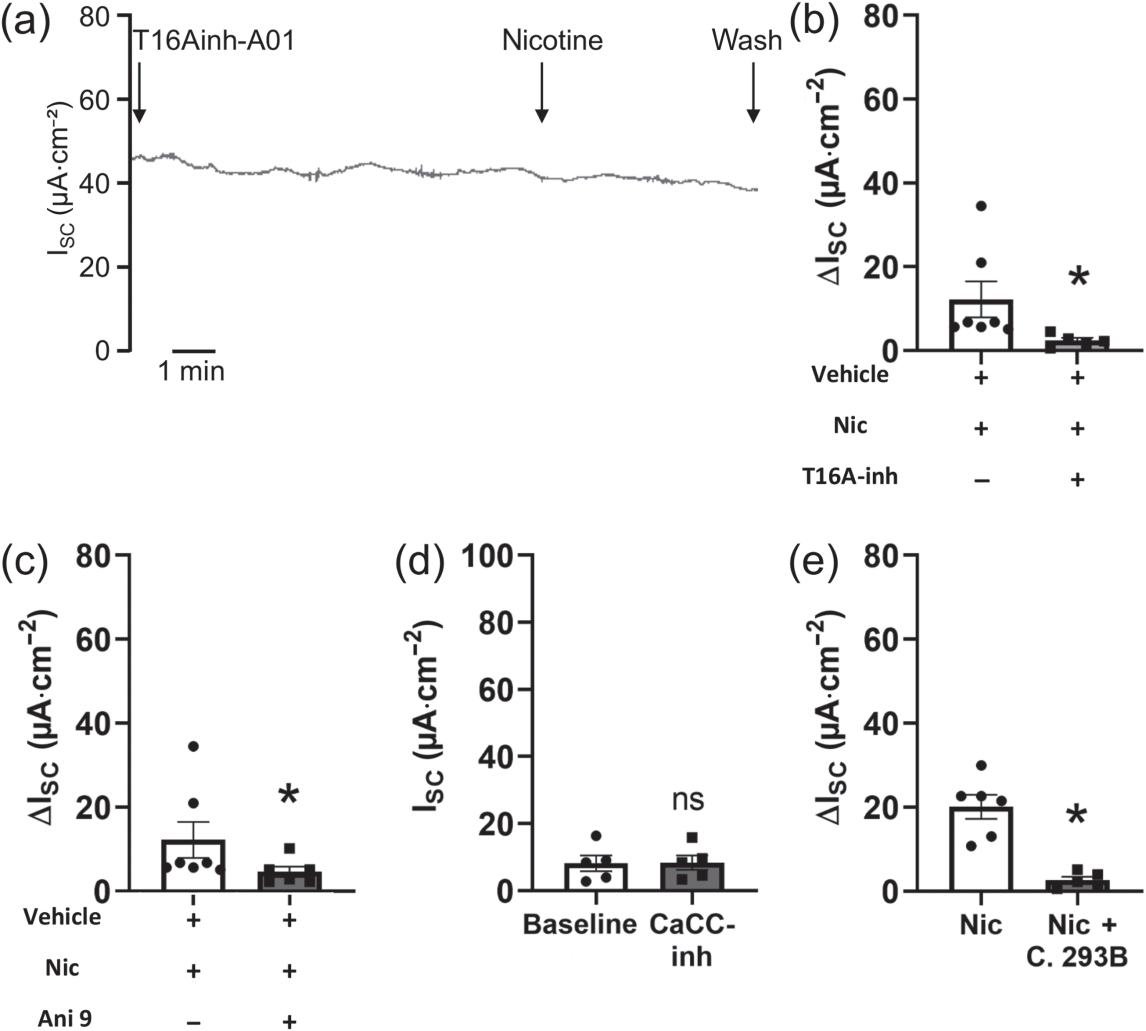
Role of chloride and potassium channels in the nicotine effect. (a) In the presence of the TMEM16A inhibitor T16Ainh‐A01, the nicotine effect was reduced. Representative current trace. (b) Apical application of T16Ainh‐A01 (10 μM) significantly reduced the nicotine effect (100 μmol·L^−1^, apical, ΔI_SC_, *P < 0.05, *n* = 5). (c) In the presence of the TMEM16A inhibitor Ani 9 (10 μmol·L^−1^, apical), the nicotine effect (100 μmol·L^−1^, apical, ΔI_SC_, *P < 0.05, *n* = 5) was significantly reduced. (d) In the presence of the inhibitor of calcium‐activated chloride channels CaCCinh‐A01 (100 μmol·L^−1^, apical), nicotine (100 μmol·L^−1^, apical) had no significant effect on I_SC_ (*n* = 5; ns, not significant). (e) Basolateral application of the KCNQ1 inhibitor chromanol 293B (C. 293B, 100 μmol·L^−1^) significantly reduced the nicotine effect (100 μmol·L^−1^, apical, ΔI_SC_, *P < 0.05, *n* = 5)

Our previous study (Hollenhorst, Lips, Weitz, et al., 2012) indicated an involvement of Ca^2+^‐dependent chloride channels in the nicotine effect. However, the exact identity of the activated chloride channel responsible for the apical chloride secretion remained elusive. Here, we performed experiments targeting the calcium‐activated chloride channel (CaCC) TMEM16A with the inhibitor T16Ainh‐A01 (10 μmol·L^−1^, Namkung, Phuan, & Verkman, 2011). In the presence of T16Ainh‐A01 apically, the nicotine effect was significantly reduced by 80% (Figure 6a,b). Also, another TMEM16A inhibitor Ani 9 (10 μmol·L^−1^, apical) reduced the nicotine effect significantly by 77% (Figure 6c). In the presence of CaCCinh‐A01 (100 μmol·L^−1^, apical), an inhibitor of CaCCs, the nicotine effect was completely abolished (Figure 6d). These results identify the contribution of CaCCs and specifically TMEM16A to the nicotine effect. Due to evidence that PKA mediates in the nicotine effect (Figure 4d), we also addressed the role of the PKA‐activated potassium channel KCNQ1. Inhibition of KCNQ1 with chromanol 293B (100 μmol·L^−1^, basolateral) significantly reduced the nicotine effect by 87% (Figure 6e). This indicates that the nicotine‐induced apical chloride secretion is driven by activation of the basolateral potassium channel KCNQ1.

## 4 DISCUSSION

We have recently shown that ACh plays an important role as a non‐neuronal autocrine and paracrine signalling molecule by increasing mucociliary clearance in the mouse tracheal epithelium in response to bacterial molecules (Hollenhorst et al., 2020). This is of considerable importance and clinically relevant, as this mechanism provides an effective response to remove inhaled pathogens. Using Ussing chamber experiments, we have previously shown that in the mouse tracheal epithelium functional nAChRs are expressed and that their activation leads to a transient apical chloride secretion which is dependent on a basolateral potassium secretion (Hollenhorst, Lips, Weitz, et al., 2012). In the present study we have confirmed the findings that application of nicotine leads to a transient current increase, observing an EC_50_ of 19.83 μmol·L^−1^. This is in agreement with observations in monkey bronchial epithelial cells, where the EC_50_ for nicotine was 26.5 μmol·L^−1^ (Xiao, Lindstrom, & Spindel, 2009). Whereas in recombinant human nAChR overexpressing *Xenopus* oocytes, EC_50_ values for nicotine had a broad range from the lowest EC_50_ of 5.02 μmol·L^−1^ for α4β4 nAChR to the highest EC_50_ of 132.44 μmol·L^−1^ for α3β2 nAChR (Chavez‐Noriega et al., 1997), showing that our EC_50_ is well in this range.

Our observation that α‐bungarotoxin, α‐conotoxin ImI and ACV‐1, antagonists for α7, α9 and α9α10 nAChR, did not alter the nicotine effect shows that these nAChR subtypes are not involved in the nicotine effect and that rather mixed αβ heteromeric receptors are responsible for the effect. Nevertheless, the α7 nAChR is expressed in mouse tracheal epithelium as we have shown here and previously (Hollenhorst, Lips, Weitz, et al., 2012). This receptor was found to influence transepithelial ion transport by other mechanisms as it was functionally coupled to cystic fibrosis transmembrane conductance regulator activation (Maouche et al., 2013). The complete inhibition of the nicotine effect by pretreatment with the non‐selective nAChR antagonist mecamylamine observed in present study supports the conclusion that the nicotine‐induced effect was indeed due to activation of heteromeric αβ nAChRs. Consistent with these observations, desensitization was observed upon a second agonist application for the αβ heteromeric nAChRs but not for the α7 nAChR (Chavez‐Noriega et al., 1997).

Indeed, the α3β2, α4β2, α4β4 and α3β4 nAChR agonist epibatidine and the α4β2 and α3β4 agonist A‐85380 mimicked the nicotine effect in our study. In accordance with a study from Sullivan et al. (1996), A‐85380 was more potent than nicotine but less potent than epibatidine. Dihydro‐β‐erythroidine, a competitive antagonist of α4β2, α3β2, α4β4 and α3β4 nAChRs, significantly reduced the nicotine effect in a concentration of 10 μmol·L^−1^. The affinity of the antagonist varies for the different nAChR subtypes with IC_50_ values of 1.3 μM for the α2β2 nAChR, 2.3 μM for the α2β4 nAChR, 0.41 μM for the α3β2 nAChR, 23.1 μM for the α3β4 nAChR, 0.37 μM for the α4β2 nAChR and 0.19 μM for the α4β4 nAChR (Harvey & Luetje, 1996). The residual effect observed at 10 μmol·L^−1^ and the fact that besides α3β4 nAChRs all other α3 or α4 containing nAChR subtypes should have already been blocked at a concentration of 1 μmol·L^−1^ point towards the α3β4 nAChR being responsible for the nicotine effect. Also, our observation that the α3 subunit was present in almost all epithelia analysed by PCR points to the α3 subunit as being responsible for the nicotine effect. Supportively, the α3β2 antagonists, α‐conotoxin MII and α‐conotoxin PnIA only attenuated the nicotine effect in doses much higher than their IC_50_ of 0.5 nM for MII (Cartier et al., 1996) and 9.56 nM for PnIA (Luo et al., 1999), indicating that they might also act on similar nAChR, such as the α3β4 receptor. This is supported by our findings in β2^−/−^ and β4^−/−^ nAChR mice that clearly demonstrate the β4 subunit is responsible for the nicotine effect. The complete abolishment of the nicotine effect in β4^−/−^ nAChR mice is not due to reduced reaction caused by a deterioration of epithelial integrity as the epithelia still reacted to ATP that was used to finale experiments as a viability control (data not shown). Taken together, these results show that the α3β4 nAChR is the subtype that activates transepithelial ion transport in the mouse tracheal epithelium. In support of this, this receptor subtype has recently been shown to transiently activate ciliary beat (Perniss, Latz, et al., 2020), indicating that it is essential for the regulation of both components of mucociliary clearance, ciliary beat and airway surface liquid regulation.

Additionally, in the present experiments we have elucidated the nAChR downstream signalling cascades leading to the nicotine effect. Previously, we have shown that the nicotine effect in the mouse tracheal epithelium is mediated only to a small extend by Ca^2+^ influx from extracellular sources into the cell and Na^+^ influx played no role (Hollenhorst, Lips, Weitz, et al., 2012). In rat colonic epithelium, metabotropic nAChRs that activate Na^+^/K^+^‐ATPase currents have been reported (Bader, Lottig, & Diener, 2017; Lottig, Bader, Jimenez, & Diener, 2019) revealing a mode of action that is independent from an ionotropic action.

Our observation that the nicotine effect was reduced when the release of intracellular Ca^2+^ from the intracellular stores was inhibited by thapsigargin underlines the involvement of metabotropic nAChRs that act via the release of Ca^2+^ from the endoplasmatic reticulum rather than forming ligand‐gated ion channels. Additionally, our Ca^2+^ imaging results clearly show that stimulation with nicotine leads to a [Ca^2+^]_i_ increase in tracheal epithelial cells due to a release of Ca^2+^ from intracellular stores, since the same percentage of cells reacted to nicotine independently of the extracellular Ca^2+^ concentrations. Furthermore, our results with the RyR antagonists, JTV519 and dantrolene (Hunt et al., 2007; Zhao, Li, Chen, Louis, & Fruen, 2001), demonstrate that Ca^2+^ is released from the endoplasmatic reticulum via RyR. Taken together, these results provide first evidence for metabotropic nAChR signalling in mouse tracheal epithelium. Interestingly, the nicotine effect seems to be mediated via RyR as well as IP3 receptors as we have previously shown (Hollenhorst, Lips, Weitz, et al., 2012). RyR and IP3 receptors are structurally related but are generally assumed to have different physiological profiles (Santulli, Nakashima, Yuan, & Marks, 2017).

Our findings of metabotropic nAChR add to the increasing evidence for metabotropic nAChR signalling (Kabbani & Nichols, 2018). In rat colonic epithelial cells, Lottig, Bader, Jimenez, and Diener (2019) recently found that nAChRs activate the Na^+^/K^+^ATPase via cytosolic increased Ca^2+^ and PKC. However, in the tracheal epithelium, we could not find evidence for the involvement of PKC in response to nicotine. Further, evidence for metabotropic activity of nAChRs interfering with changes of [Ca^2+^]_i_ was found in rat alveolar macrophages (Mikulski et al., 2010). In these cells, nAChRs reduced ATP‐induced Ca^2+^ release and this was also independent of the presence of extracellular Ca^2+^.

**FIGURE 7:**
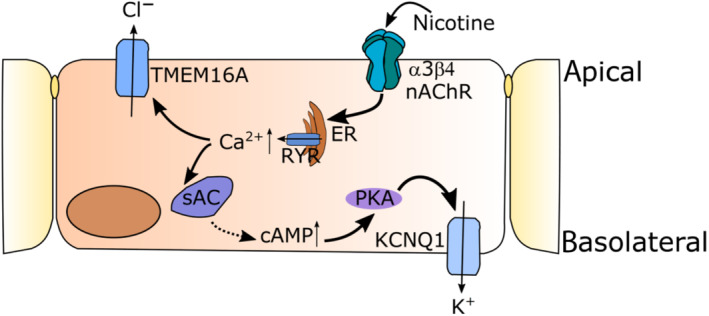
Schematic drawing of the signalling pathway delineated in our study. Activation of epithelial α3β4 nAChR by nicotine leads to a release of Ca^2+^ from the endoplasmatic reticulum (ER) via ryanodine receptors (RyR). This activates the Ca^2+^‐dependent chloride channel TMEM16A and the PKA via soluble ACs (sAC). PKA then activates the basolateral KCNQ1 potassium channel

We have previously found an involvement of adenylyl cyclase (AC) in the nicotine effect (Hollenhorst, Lips, Kummer, & Fronius, 2012). Second messenger signalling can be interconnected, for example, signalling by Ca^2+^ and cAMP, because there are calcium‐dependent ACs (Shahidullah, Mandal, & Delamere, 2017). Here, we showed for the first time in non‐neuronal cells that nAChRs activate PKA, as the PKA inhibitor H‐89 (Chijiwa et al., 1990) attenuated the nicotine effect. So far, this has only been described for neurons, where nicotine acts on γ oscillations in the rat hippocampus via PKA (Wang et al., 2017). PKA involvement in the nicotine effect (Figure 7) is further supported by our findings that KCNQ1 is involved in the nicotine effect, as this channel is a phosphorylation target of PKA downstream of cAMP activation (Marx et al., 2002).

Effectors of nAChR activation in mouse tracheal epithelium are channels involved in chloride secretion (Hollenhorst, Lips, Weitz, et al., 2012), but the type of channels involved remains unknown. Interestingly, in the present study we observed the activation of the Ca^2+^‐activated chloride channel TMEM16A via nAChR signalling. This is of particular importance, because targeting this channel has been discussed as an alternative drug target to restore Cl^−^ secretion in cystic fibrosis patients due to defective cystic fibrosis transmembrane conductance regulator function (Danahay et al., 2020). The observed transient increase in TMEM16A observed in our study might also be beneficial in cystic fibrosis, since one study hypothesized that a transient stimulation of chloride secretion is impaired in cystic fibrosis (Song et al., 2009). Interestingly, non‐neuronal cholinergic signalling was down‐regulated in cystic fibrosis patients (Wessler et al., 2007), further underlining the putative beneficial effects of targeting the nAChR signalling in airway epithelia, as investigated in our study.

Taken together, our study identifies α3β4 nAChRs as the main subunits responsible for the nicotine effect. The activation of which leads to an increase of [Ca^2+^]_i_ released from the endoplasmatic reticulum and PKA activation. This [Ca^2+^]_i_ increase in turn activates the TMEM16A chloride channel. These nAChRs might represent a novel pharmacological target to restore defective anion secretion in conditions such as cystic fibrosis, chronic obstructive pulmonary disease and cigarette smoke‐induced chronic bronchitis and to improve mucociliary clearance.

## AUTHOR CONTRIBUTIONS

P.K. and G.K.‐C. performed the experiments. P.S. kindly provided the access to the transgenic animals. P.K., M.I.H. and G.K.‐C. wrote the manuscript and interpreted the data. M.I.H. and G.K.‐C. designed the study. P.S., M.F., M.I.H. and G.K.‐C. critically reviewed the manuscript. All authors approved the final version of the manuscript.

## CONFLICT OF INTEREST

The authors declare no conflicts of interest.

## DECLARATION OF TRANSPARENCY AND SCIENTIFIC RIGOUR

This Declaration acknowledges that this paper adheres to the principles for transparent reporting and scientific rigour of preclinical research as stated in the *BJP* guidelines for Design & Analysis and Animal Experimentation, and as recommended by funding agencies, publishers and other organizations engaged with supporting research.
